# Genetic and Molecular Analyses of *PEG10* Reveal New Aspects of Genomic Organization, Transcription and Translation

**DOI:** 10.1371/journal.pone.0008686

**Published:** 2010-01-13

**Authors:** Heike Lux, Heiko Flammann, Mathias Hafner, Andreas Lux

**Affiliations:** 1 Institute of Molecular and Cell Biology, Mannheim University of Applied Sciences, Mannheim, Germany; 2 Faculty of Medicine at Mannheim, University of Heidelberg, Mannheim, Germany; Duke University, United States of America

## Abstract

The paternally expressed gene PEG10 is a retrotransposon derived gene adapted through mammalian evolution located on human chromosome 7q21. PEG10 codes for at least two proteins, PEG10-RF1 and PEG10-RF1/2, by -1 frameshift translation. Overexpression or reinduced PEG10 expression was seen in malignancies, like hepatocellular carcinoma or B-cell acute and chronic lymphocytic leukemia. PEG10 was also shown to promote adipocyte differentiation. Experimental evidence suggests that the PEG10-RF1 protein is an inhibitor of apoptosis and mediates cell proliferation. Here we present new data on the genomic organization of PEG10 by identifying the major transcription start site, a new splice variant and report the cloning and analysis of 1.9 kb of the PEG10 promoter. Furthermore, we show for the first time that PEG10 translation is initiated at a non-AUG start codon upstream of the previously predicted AUG codon as well as at the AUG codon. The finding that PEG10 translation is initiated at different sides adds a new aspect to the already interesting feature of PEG10's −1 frameshift translation mechanism. It is now important to unravel the cellular functions of the PEG10 protein variants and how they are related to normal or pathological conditions. The generated promoter-reporter constructs can be used for future studies to investigate how PEG10 expression is regulated. In summary, our study provides new data on the genomic organization as well as expression and translation of PEG10, a prerequisite in order to study and understand the role of PEG10 in cancer, embryonic development and normal cell homeostasis.

## Introduction

In 2004, the International Human Genome Sequencing Consortium published an analysis and annotation of the nearly completed human genome sequence (∼99%) with an estimated number of 20.000–25.000 protein coding genes [1]. Despite this surprisingly low number of coding genes the complexity of the proteome is generated in part by alternative splicing. Alternative splicing gives rise to a varying number of mRNAs coding for a set of one to several differentially assembled proteins originating from one gene. The imprinted human gene “Paternally Expressed Gene” 10 (*PEG10*) and its mouse ortholog *Peg10/Edr* use a different mechanism coding for more than one protein by −1 ribosomal frameshift translation [2], [3], which is well known from retroviruses and retrotransposons [4]. *PEG10* and the human paraneoplastic antigen gene *MA3* [5] are to our knowledge currently the only two human genes known to use this mechanism.

*PEG10* that is thought to be derived from the Ty3/Gypsy family of retrotransposons [6] has two open reading frames named RF1 and RF2 overlapping by 61 nucleotides. RF1 codes for the gag-like PEG10-RF1 protein and RF2 codes for a pol-like protein and is part of the PEG10-RF1/2 fusion protein due to −1 frameshift translation. PEG10-RF1 contains an N-terminal coiled-coil domain and a C-terminal zinc finger domain, commonly found in retroviral *gag* proteins. In addition, there is a functional aspartic protease motif in PEG10-RF1/2 shortly after the frameshift site leading to proteolytic cleavage products of different sizes [2], [7]. In order to perform the −1 frameshift, the RF1-RF2 overlap sequence contains a seven nucleotide “slippery” sequence with typical consecutive homopolymeric triplets. The underlined PEG10 “slippery” heptanucleotide sequence *GGGAAACTC* follows the general pattern of *X XXY YYZ* where the A- and P-site tRNAs detach from the zero frame codons *XXY YYZ* and re-pair after shifting back one nucleotide to *XXX YYY* (4). Thus, the deduced amino acid sequence of the frameshift site after frameshift translation is GNGKL (translated nucleotide sequence: *GGA AAC GGG AAA CTC*). The spacer region between the shift site and the 3′ pseudoknot that in addition to the “slippery” heptanucleotide is necessary to promote the −1 frameshift is 5 nucleotides long [8].

*PEG10* orthologs were identified in 11 other eutherian species as well as in the metatherian tammar wallaby (*Macropus eugenii*) [7], [9], [10], [11], [12]. The heptanucleotide “slippery” sequence is completely conserved in all species and the sequence of the downstream pseudoknot is completely conserved in the mammalian species too, except for one nucleotide change in the rodent sequence [7]. This conservation suggests that all mammalian species have retained the ability to translate both *PEG10* reading frames by −1 frameshifting. Within the group of eutherian *PEG10* orthologs, mouse and rat *Peg10* are the most divergent from the others and contain small and large insertions within both reading frames, i.e. a large insertion within the second reading frame of the mouse gene [2], [11].

The adaptation and fixation of a former retrotransposon in the genome and its high conservation in different mammalian species argues for an important function of PEG10. Studies with mice showed a high expression during embryonic development especially from day 9.5 to 16.5., specifically in bone and cartilage forming tissues. High expression was also seen in extra embryonic tissues at all stages between E7.5 and E17.5. Mouse placenta is positive for *Peg10* transcripts as well as the Peg10-RF1 and Peg10-RF1/2 proteins [7]. *Peg10* knock-out mice showed early embryonic lethality at 10 days post-coitus due to defects in the placenta [13]. In addition, mouse *Peg10* is highly expressed in the embryo but so far only detected in testis and brain of adult animals [3]. In humans, high expression of *PEG10* in adult tissues was seen in brain, kidney, lung, testis and weak expression in spleen, liver, colon, small intestine and muscle [2], [9]. *PEG10* expression in human placenta and detection of the corresponding PEG10-RF1 and PEG10-RF1/2 proteins was shown for different gestation stages [7], [14].

PEG10 is not only involved in embryonic development but *PEG10*-induced expression was also reported for regenerating mouse liver [15]. Furthermore, overexpression or reinduced expression of *PEG10* was seen in several malignancies, like hepatocellular carcinoma [15], [16], [17], the embryonic kidney malignancy Wilms tumor (WT) [18], pancreatic cancer [19], B-cell acute and chronic lymphocytic leukemia (ALL and CLL) [20], [21] and the embryonic form of biliary atresia [22]. It appears that overexpression of *PEG10* leads to an inhibition of apoptosis in hepatic cancer [16] and B-cell ALL and CLL [20], [21], which was demonstrated by either siRNA mediated knock-down of PEG10 leading to apoptosis or by overexpression of the PEG10-RF1 protein in hepatoma cells rendering resistance to apoptosis and promoting cell growth [16]. It was further shown, that PEG10 binds to different TGF-β receptors and blocks TGF-β signaling [2].

The *PEG10* gene consists of two exons, separated by a 6.8 kb intron, giving rise to a ∼6.6 kb transcript [2]. Exon 1 contains the 5′-UTR and exon 2 contains the RF1 and RF2 coding regions and a ∼4 kb 3′-UTR sequence. *PEG10* is located on human chromosome 7q21 in a head-to-head orientation with the also paternally expressed sarcoglycan ε gene *SGCE*. Both genes belong to an imprinted gene cluster in humans as well as in the syntenic chromosomal regions of several other mammalian species [23], [24], [25], [26]. Based on the human genome sequence, *PEG10* and *SGCE* are separated by less than 200 bps. Previously, it was reported that the male hormone androgen promotes *PEG10* expression and that the androgen receptor directly interacts *in vivo* with androgen-responsive elements in the promoter region and exon 2 of the *PEG10* gene [27]. In a different study it was shown that c-MYC enhances *PEG10* expression in solid cancer and B-cell lymphoblasts [19]. There are several conflicting data entries (AB028974, AB049150, AB049834, AF216076, AL589326, BC050659, BP250746) with various PEG10 5′-UTR mRNA sequence lengths. Thus, the exact transcription start site is uncertain. Therefore, in line with this, the exact *PEG10* promoter region is not clear either.

In the context of cancer it is important to know how *PEG10* expression and translation is regulated and what promoter regions are involved. One goal of this study was to analyse the *PEG10* promoter region. Firstly, the major transcription start site (mTSS) was defined as well as the existence of further upstream located TSSs and secondly, a 2 kb fragment upstream of the mTSS was cloned. The promoter activities of this 2 kb sequence as well as smaller fragments were analysed using luciferase reporter assays and bioinformatics. We also investigated the effect of the *PEG10* 5′-UTR sequence on PEG10 translation and show that *PEG10* uses different translation start sites than the one previously predicted. Furthermore, our analyses led to the identification of a *PEG10* splice product containing a putative new AUG start codon located in frame upstream of the previous one.

## Results

### Identification of the PEG10 Major Transcription Start Site

**Figure 1:**
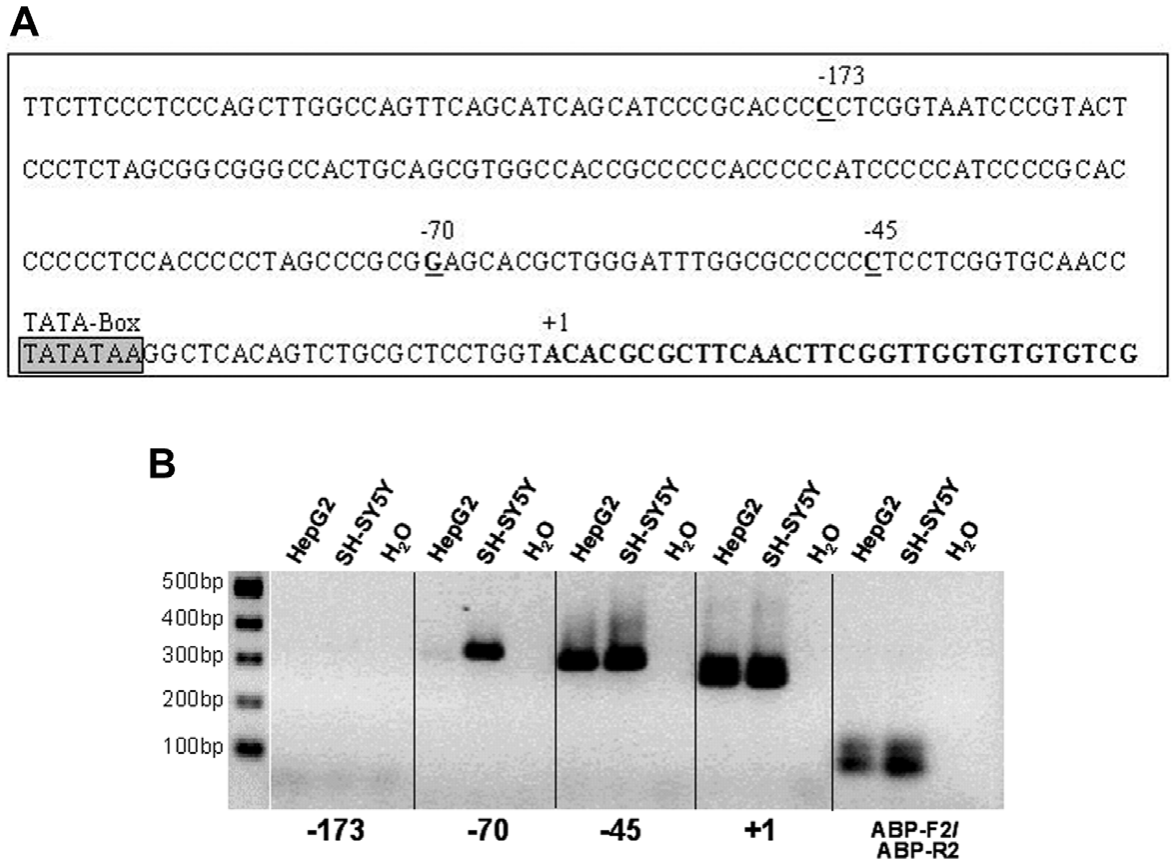
The *PEG10* major transcription start site and alternative start sites. A. Upstream and downstream sequence around the major transcription start site (mTSS) numbered +1. The positions −45, −70 and −173 represent binding sites of primers used to analyse whether alternative TSSs exist located further upstream. In addition, at positions −79 and −112, a T/G and T/C polymorphism, respectively, was identified (data not shown). B. Total RNA of the indicated cell lines were reversely transcribed with a *PEG10*-specific oligonucleotide (KIAA-3′INNER) located in exon 2. The cDNA was then used in a PCR reaction with the *PEG10* exon 2-specific reverse primer ABP-R2 (located upstream of KIAA-3′INNER) and forward primers located at the indicated positions in regard of the mTSS (see A.). The ABP-F2 oligonucleotide is located in exon 1 next to the exon1|intron border.

In order to determine the exact *PEG10* transcription start site (TSS) we performed 5′-RACE experiments with RNA from the hepatoma cell line HepG2, previously reported to express *PEG10* [2], [15], [16], and the neuroblastoma cell line SH-SY5Y. PEG10 was shown to be highly expressed in brain [2], [9]. Therefore, we speculated that PEG10 might be expressed in SH-SY5Y cells, which was indeed the case when tested for by semi-quantitative RT-PCR (data not shown). For the 5′-RACE experiments HepG2 and SH-SY5Y RNA was reverse transcribed with a *PEG10*-specific primer located approximately 500 bp away from the putative 5′-end of the *PEG10* transcript, which was chosen to increase the chance for complete extension to the 5′ -end of the message. The *PEG10* 5′-RACE PCR products were subsequently cloned into a vector and insert sequences were analysed by sequencing. All clones that contained a PEG10 specific insert started with an expected polymeric run of Gs (complementary sequence of the adapter) and continued with ACACGCGCTTCAACT… . On the genomic level, 24–30 nucleotides upstream of this sequence, there is a typical TATA-box sequence (Figure 1A). We concluded that this sequence most likely represents the TSS and numbered the A +1 corresponding to nucleotide position 19519958 of reference sequence NT_007933|Hs7_8090.

The literature and database entries suggest additional TSSs located further upstream, for example at position −45. Therefore, we used the 5′RACE products in a PCR with *PEG10* reverse primer ABP-R2 (located in exon 2 close to the splice site junction) and a primer for the −45 position. The subsequent PCR resulted in amplification products of the expected size for HepG2 as well as SH-SY5Y cells (Figure 1B). Next, we tested whether there are *PEG10* transcripts starting at even further upstream positions including −70 and −173. No products were seen for the PCR with the –173/ABP-R2 primer pair. PCR with primer pair –70/ABP-R2 was positive for SH-SY5Y whereas only a very faint band of the expected size was seen with the HepG2 5′RACE template (Figure 1B).

These results suggest (a) that HepG2 and SH-SY5Y cells use alternative TSSs from the one predicted in the initial experiment and (b) that the use and strength of alternative *PEG10* TSSs differs between these two cell types at position −70. Nevertheless, due to the fact that we used gene-specific 5′-RACE products in a semi-nested PCR approach this might have led to an artificial over representation of alternative *PEG10* transcripts in relation to the suggested major TSS. Therefore, we tested different cell lines for *PEG10* expression and transcription start sites by semi-quantitative RT-PCR. Two different primer pairs were used with an identical reverse primer (ABP-R2) but variable forward primers specific for transcription start sites at position +1 or −45, respectively. For this analysis the cDNA of various cell lines were employed in the PCRs.

**Figure 2:**
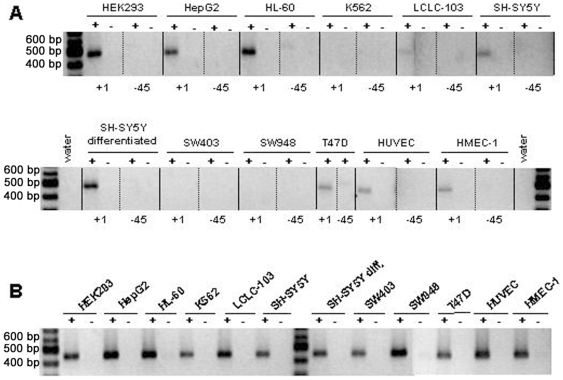
Analysis of *PEG10*-specific transcription and transcription start sites. *PEG10* expression and transcription start sites were analysed by semi-quantitative RT-PCR. **A.** Total RNA of the indicated cell lines was reverse transcribed (RT) with a mixture of oligo-dT/hexamer oligonucleotides. The cDNAs were used in PCR reactions with an identical PEG10-specific reverse primer (KIAA-3′INNER) but variable forward primers specific for transcription start sites at position +1 or −45, respectively, resulting in approximately 500 bp products. **B.** PCRs for the GAPDH gene served as a positive control for the RT reaction and the use of equal amounts of template RNA. +, PCR with cDNA; −, PCR with total RNA.

PEG10 is expressed in the cancer cell lines HepG2, HL-60 (promyelocytic leukaemia-derived cells), LCLC-103H, T47D and the neuroblastoma-derived SH-SY5Y cells as well as in HEK293 and endothelial cells like primary HUVECs and HMEC-1 cells (Figure 2A). No expression was detected for the myelogenous leukemia cells K562 and the colon cancer cell lines SW403 and SW948. Second, the data show that our identified TSS is indeed the major TSS since the PCR with the +1 TSS-specific forward primer produces a strong amplification product for all PEG10 positive cells. Whereas, with the −45 TSS-specific forward primer less intense PCR products were only seen for HL60 and T47D cells (Figure 2A) and in addition only very faint or almost invisible bands were seen for HEK293, and SH-SY5Y differentiated cells. Furthermore, it was observed that the PEG10 expression level between the different cell types varies. PCRs for the housekeeping gene GAPDH served as a positive control for the RT reactions and the use of equal amounts of cell line-specific template RNA (Figure 2B). It is interesting to note that differentiated SH-SY5Y cells show a stronger expression than non-differentiated cells. In a separate experiment we followed-up on this and found that PEG10 expression increases during the differentiation process (see Materials and Methods, data not shown).

### Variable PEG10 Transcript Length Due to Alternative Polyadenylation

**Figure 3:**
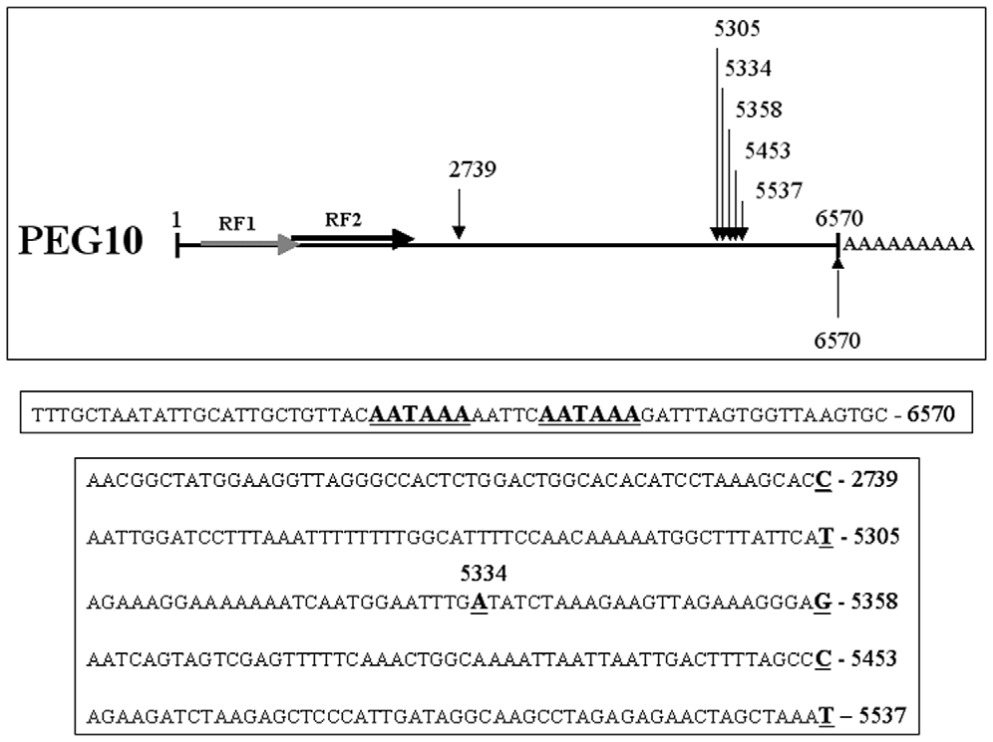
*PEG10* polyadenylation. Shown is a schematic view of the 6570 bp long *PEG10* transcript with a poly-A tail at its 3′-end. Shown is also the *PEG10* mRNA 3′-end sequence with the two underlined canonical polyadenylation signal motifs. The arrows and appendant numbers mark the nucleotide positions of putative alternative polyadenylation sites according to identified *PEG10* transcripts. The sequences preceding the polyadenylated nucleotides do not contain a canonical polyadenylation signal motif.

By Northern Blot analysis it has been shown previously that *PEG10* transcripts of different size exist [2], [9]. Therefore, we isolated the 3′-ends of *PEG10* transcripts by reverse transcription of HepG2 and SH-SY5Y total RNA with an Oligo-dT-Clamp oligonucleotide generating cDNAs starting at the 3′-ends of gene transcripts. The resulting cDNA was used in a PCR with different 3′-UTR specific *PEG10* forward primers in combination with a Clamp specific reverse primer (3′-RACE). By this approach, in addition to the major polyadenylated ∼6.6 kb transcript, alternatively polyadenylated transcripts of 2.7 kb and 5.3–5.5 kb in length were detected. However, none of these transcripts contained the typical “AATAAA” polyadenylation signal motif at their 3′-end (Figure 3).

### Cloning and Analysis of the *PEG10* Promoter

**Figure 4:**
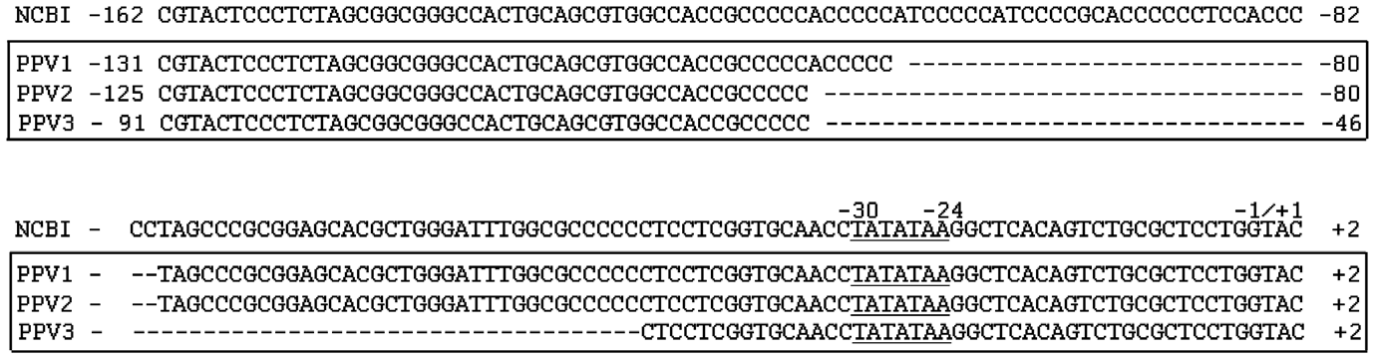
*PEG10* promoter variants. The sequence named NCBI −162 to +2 corresponds to nucleotides 19518795 to 19519959 of reference sequence NT_007933|Hs7_8090 and represents the PEG10 promoter sequence in regard to the *PEG10* mTSS. Analyses of this promoter region revealed three different *PEG10* promoter variants (PPV) named PPV1, 2 and 3. The variants are characterised by 31, 37 and 71 nucleotide deletions as compared to the reference sequence.

**Figure 5:**
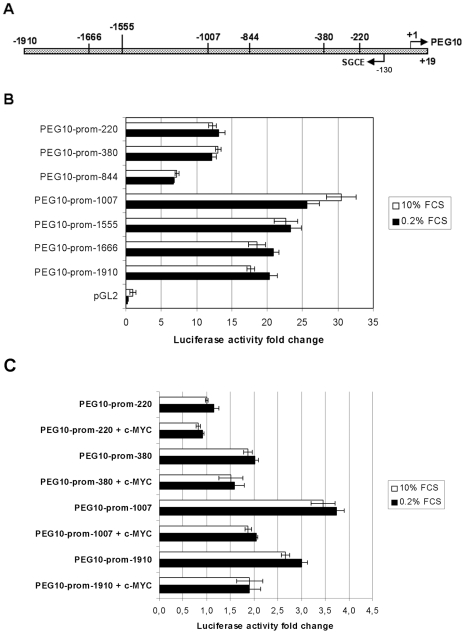
Analysis of *PEG10* promoter fragments by promoter-reporter luciferase assays. A. Shown is a schematic view of 1.9 kb of the *PEG10* promoter variant 1 (PPV1). The numbers indicate the 5′-end and length in bp of the evaluated promoter regions in (B.) and (C.) in relation to the transcription start site +1. B. Functional analysis of the *PEG10* promoter. *PEG10* promoter regions of different length, as indicated, were cloned upstream of the luciferase gene into the reporter plasmid pGL2-Basic. Equal molar amounts of the different promoter constructs were transfected into HEK293 cells. Luciferase activity was measured 48h after transfection and an over-night cultivation with either 10% FCS or 0.2% FCS. The data represent the mean of two independent experiments performed in triplicates plus standard deviation and normalized for transfection efficiency. **C.** Ectopic c-MYC expression inhibits *PEG10* promoter activity. HEK293 cells were co-transfected with a c-myc expression construct and equal molar amounts of the indicated promoter constructs. Luciferase assays were performed as described for (B.). The activity of the PEG10-prom^−220^ reporter in the presence of 10% FCS was set to one and used as a reference point to calculate the relative activity changes. The data represent the mean of two independent experiments performed in triplicates plus standard deviation and normalized for transfection efficiency.

In order to identify and analyse the *PEG10* promoter, an approximately 2 kb genomic DNA fragment was amplified from genomic DNA isolated from peripheral blood mononuclear cells with primers located according to our TSS results at position –1941 and position +19. This fragment corresponds to nucleotides 19518017 to 19519976 of reference sequence NT_007933|Hs7_8090. The putative *PEG10* promoter region was cloned upstream of the firefly luciferase gene into the reporter plasmid pGL2-Basic. Surprisingly, the cloned promoter region contained a 31 nucleotide deletion compared to the human reference sequence (nucleotide position 19519848-19519878) (see Figure 4). Therefore, we went back and sequenced several more clones containing the insert sequence with no different result. Because the deleted sequence has a repeat structure, (CCCCCN)_x_, we initially assumed that the sequence was deleted due to bacterial recombination activities. Consequently, we repeated the promoter fragment cloning obtaining identical results. This suggested that the deletion is indeed on the genomic level. Nevertheless, we could not completely exclude the possibility that recombination events take place in the bacteria despite the use of a *recA* ^−^ strain. Therefore, we amplified this region from HEK293 genomic DNA and from ten individuals with a European or US-American genetic background. The sequences were determined directly from the PCR products by bi-directional analysis. This confirmed the 31 bp deletion. In addition, we identified 37-bp and 71-bp deletions at nucleotide positions 19519842-19519878 and 19519842-19519912, respectively. HEK293 cells have a homozygous 71 bp deletion whereas most of the individuals were heterozygous for the 31/37 bp deletions or in one case for a 31/71 bp deletion. Therefore, in contrast to the rest of the sequenced promoter region, none of the individual genomic sequences analysed conform to the human reference sequence at the investigated position. We named the region with the 31-bp deletion *PEG10* Promoter Variant 1 (PPV1), with the 37-bp deletion PPV2 and with the 71-bp deletion PPV3. The initially cloned fragment represents a 1910 bp long *PEG10* promoter region upstream of the TSS. This construct was named PEG10-prom^−1910^ and served as the template for further shortened *PEG10* promoter constructs (see Figure 5).

Next, we tested the promoter activity of the –1910 bp region and six additional shorter promoter constructs in HEK293 cells since HEK293 cells express *PEG10* endogenously. Figure 5A shows a schemetical view of the analysed region. The highest reporter activity was observed for the PEG10-prom^−1007^ construct (Figure 5B and 5C). The lowest activity was always seen for PEG10-prom^−844^ followed by PEG10-prom^−220^ and PEG10-prom^−380^. Figure 5B shows the mean of two independent experiments each performed in triplicate. These results were obtained under standard medium conditions in the presence of 10% FCS. It was previously reportet that *PEG10* expression is up regulated by serum deprivation [28]. Therefore, we analysed the promoter activity under low serum conditions by incubating the cells in the presence of 0.2% FCS for 16 hours. The activity of the promoter constructs were either unchanged or slightly up regulated under low serum concentrations with the exception of PEG10-prom^−1007^. Serum deprivation led to a slight but consistent increase in PEG10-prom^−1910^ reporter activity in all assays (Figure 5B and 5C). Whereas for PEG10-prom^−1007^ 0.2% FCS led to increased (Figure 5C) as well as decreased reporter activity (Figure 5B) compared to 10% serum conditions. In conclusion, it can be said that the most 5′ 1 kb of the *PEG10* promoter sequence tested shows the highest promoter activity, and contains a repressor region between −380 and −844 and an enhancer region between −844 and −1007. Further regulatory activities can be assigned to the −1007 to −1910 sequence which showed some promoter repression.

### Influence of c-MYC on *PEG10* Promoter-Reporter Activities and Expression

It was previously reported that c-MYC up-regulates *PEG10* expression [19]. Thus, we analysed the effect of c-MYC on our promoter constructs. HEK293 cells were co-transfected witha *c-MYC* expression plasmid and different *PEG10* promoter constructs. Cells were cultured in the presence of 10% or 0.2% FCS and reporter activity was measured after 16 hours. None of the tested promoter constructs showed an increased reporter activity due to c-MYC. Quite the opposite, ectopic *c-MYC* expression reduced the promoter reporter activity for all constructs with the highest effect for PEG10-prom^−1007^ and PEG10-prom^−1910^ under all conditions. Figure 5C shows the mean results of two independent experiments performed in triplicate. In the previous report mentioned, the authors investigated their promoter-reporter constructs in hepatocellular carcinoma cells. In addition, their reporter contained a putative c-MYC binding element, an E-box consensus sequence located in the *PEG10* intron, considered to be responsible for increased PEG10 expression by c-MYC, whereas we analysed the *PEG10* promoter activity upstream of the mTSS in HEK293 cells.

**Figure 6:**
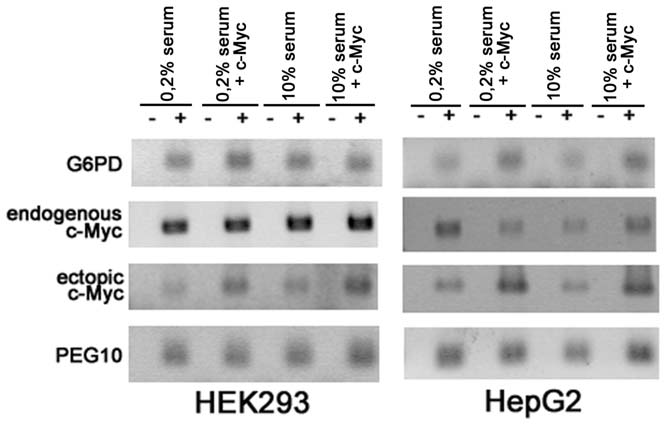
No obvious evidence for enhanced *PEG10* expression due to c-MYC. HEK293 and HepG2 cells were transfected with a *c-MYC* expression construct and then cultered over night in the presence of 0.2% or 10% FCS in parallel with non-transfected cells. Subsequently, total RNA was isolated, reverse transcribed and used for sqRT-PCR with primers specific for *G6PD*, *PEG10*, *c-MYC* (endogenous) and ectopic *c-MYC*. For further details see Results.

The use of promoter-reporter constructs in analyzing promoter activity is a legitimate and well excepted method although, only a defined shortened promoter region for a specific gene becomes analysed and consequently does not realy reflect the contextual *in vivo* situation of gene regulation with further up- or down-stream located regulatory regions. Therefore, we further analysed c-MYC regulated PEG10 expression in HEK293 and HepG2 cells by semi-quantitative RT-PCR (sqRT-PCR). HEK293 and HepG2 cells were transfected with the *c-MYC* expression construct and then cultered over night in the presence of 0.2% or 10% FCS in parallel with non-transfected cells. Subsequently, total RNA was isolated, reverse transcribed and used for sqRT-PCR with primers specific for *G6PD*, *PEG10*, *c-MYC* (endogenous) and ectopic *c-MYC*. For *c-MYC* two different primer pairs were used with an identical reverse rimer (c-MYC-rev2) but different forward primers, c-MYC-fwd2 and c-MYC-fwd. c-MYC-fwd2 binds in the 5′UTR upstream of the ATG and c-MYC-fwd, also used for expression construct cloning, binds to the ATG start codon region. Effective transfection of cells with the expression construct was expected to yield increased PCR product amounts with primer pair c-MYC-fwd/c-MYC-rev2 in comparison to non-transfected cells. This was indeed the case as shown in Figure 6. However, it appears as if there is no enhanced PEG10 expression in the presence of ectopic c-MYC.

### Detection of a *PEG10* Splice Variant

**Figure 7:**
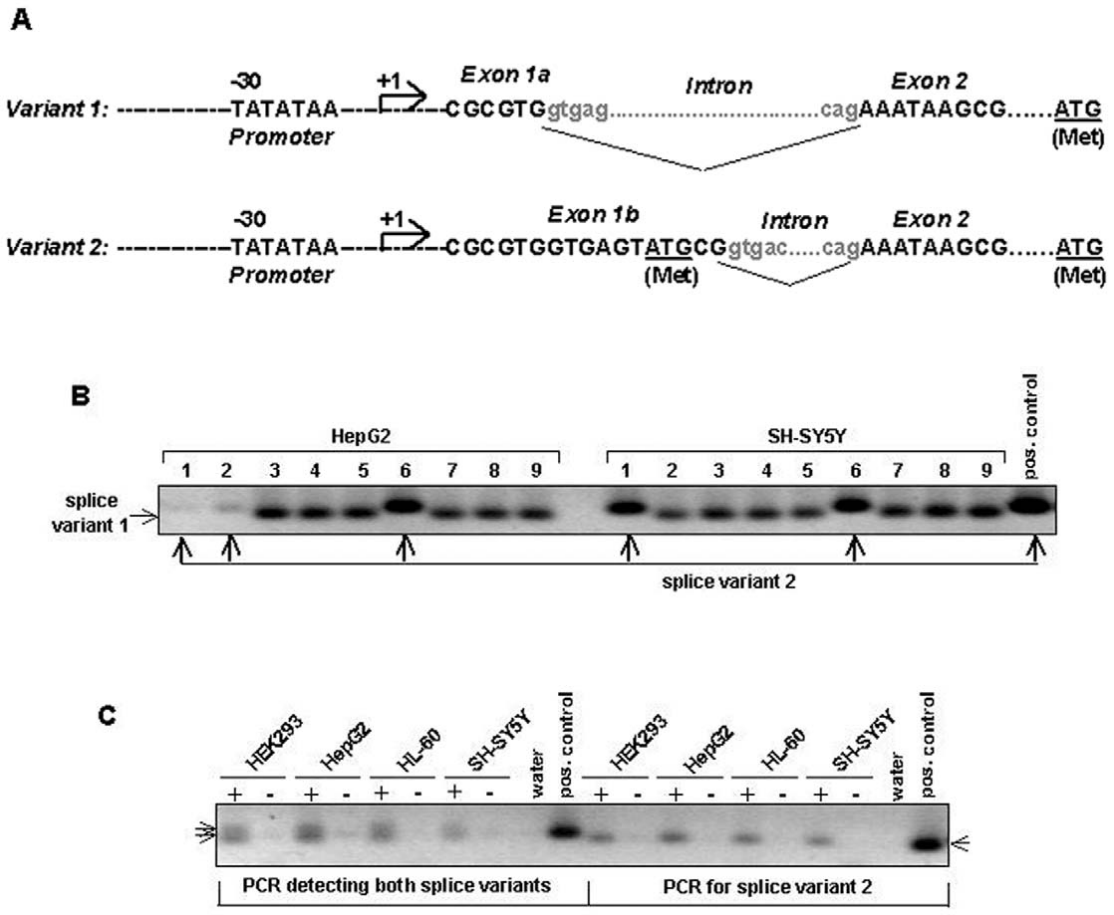
*PEG10* transcript analysis reveals the existence of two splice variants. A. Shown is the sequence around the splice junctions for the two *PEG10* splice variants. A predicted ATG start codon in exon 2 is underlined. The exon 1b transcript represents a rare splice event, which leads to a new ATG start codon (underlined) in exon 1b. B. In order to estimate the frequency of *PEG10* alternative splicing *PEG10*-specific PCR was done with cell line HepG2 and SH-SY5Y cDNA using forward and reverse primers located in exon 1 and exon 2 respectively. PCR products were cloned and transformed into bacteria. 9 colonies were analysed by colony PCR with the ABP-F2/ABP-R2 primer pair that allows to amplify both splice variants. PCR products were analysed on an agarose gel and variants were distinguished by size: Variant 1 87bp, variant 2 98bp C. PCR with splice variant specific primers shows that all of the tested cell lines express the exon 1a as well as the exon 1b transcript variants. In order to detect both splice variants (left half of Figure) or only variant 2 (right half of Figure), PCRs were performed with the following primer combinations, ABP-F2/ABP-R2 (variant 1 and 2) or ABP-F2b/ABP-R2 (variant 2 specific) respectively. +, PCR with cDNA; -, PCR with total RNA.

PEG10 consists of two exons separated by a 6.8 kb intron. When we performed the 5′-RACE experiments in order to identify the PEG10 transcription start site we used a reverse primer located in exon 2 which led to products with the exon 1/exon 2 junction sequence. Most of the products represented the expected and known exon 1/exon 2 sequence TCGCGTG/AAATAAG referred to as variant 1 or exon 1a/exon 2. However, several sequenced clones contained additional 11 nucleotides at the junction leading to the following splice product TCGCGTGGTGAGTATGCG/AAATAAG referred to as variant 2 or exon 1b/exon 2. This finding agrees with the GenBank PEG10 mRNA sequence entry AK299837.1. On the genomic level the exon 1a|intron 1 splice site sequence is TCGCGTG|gtgagtatgcg, the exon 1b|intron 1 splice site sequence is GTGAGTATGCG|gtgaggacgtt and the intron 1|exon 2 splice site sequence is tacag|AAATAA. Figure 7A shows the variant 1 and variant 2 exon/intron organization and junction sequence.

In order to analyse how frequent the alternative exon 1b splicing event occurs, we amplified this region from HepG2 and SH-SY5Y cDNAs using exon-intron-exon-spanning primers and the PCR products were cloned. Subsequently, the variant 1 or 2 specific insert sequences of 9 randomly picked clones were determined by PCR using an exon 2 located reverse primer and an exon 1a-specific forward primer, which allows the amplification of both variants with one primer set, leading to size-specific variant 1 (87 bp) or variant 2 (98 bp) products (Figure 7B). This analysis revealed the presence of splice variant 2 in three clones out of nine for HepG2 as well as two for SH-SY5Y suggesting that the exon 1b variant (variant 2) might occur with a lower frequency than the exon 1a variant (variant 1). Variant 2-specific PCR with HEK293 cDNA and HL60 cDNA, using a primer set with a forward primer specific for the variant 2 sequence leading to a 87 bp product, demonstrated that PEG10 alternative splicing is not only restricted to HepG2 and SH-SY5Y cells (Figure 7C, right part). Furthermore, we identified not only a *PEG10* splice variant but the 11 nucleotide addition includes a putative new translation start site. Splice variant 2 contains an upstream ATG, which is in frame with the in previous reports assumed ATG start codon that is located 228 bases downstream in exon 2.

### The Role of the *PEG10* 5′-UTR in Translation

**Figure 8:**
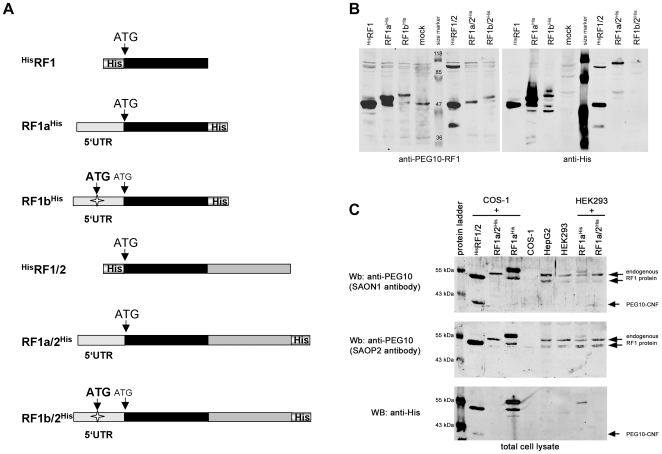
Analysis of the PEG10 5′-UTR in protein translation. A. Schematic presentation of different *PEG10* expression constructs. ^His^RF1 and ^His^RF1/2 represent constructs without the 5′-UTR and the *PEG10* coding sequence starts at the in previous reports predicted ATG start codon. The star in the 5′-UTR of the RF1b^His^ and RF1b/2^His^ constructs represent the location of a putative new ATG start codon. B. HEK293 cells were mock-transfected or with the indicated *PEG10* expression constructs. 48 h after transfection cells were lysed and aliquots of lysates were subjected to SDS-PAGE (8% gel) under reducing conditions and Western blots (Wb). PEG10 proteins were detected with PEG10-RF1-specific antibodies (SAON1/SAOP2) or an anti-His antibody. C. COS-1 and HEK293 cells were mock-transfected or with the indicated PEG10 expression constructs. 48 h after transfection cells were lysed and aliquots of lysates were subjected to SDS-PAGE (8% gel) under reducing conditions and Western blot. Lysates of non-transfected HepG2 cells were analysed in parallel. PEG10 proteins were detected with PEG10-RF1-specific antibodies (SAOP2 and SAON1) or an anti-His antibody. The size of the RF1a/2^His^ expressed PEG10-RF1 protein corresponds to the HepG2 and HEK293 (mock) endogenous PEG10-RF1 protein.

All studies published so far regarding PEG10 protein translation used expression constructs with the PEG10 RF1 or RF1/2 coding sequence starting with an ATG located in exon 2. In this context, we were interested in the effect of the *PEG10* 5′-UTR sequence on translation. For this purpose, C-terminal His-tagged PEG10-RF1 and PEG10-RF1/2 expression constructs were generated for both splice variants of the *PEG10* 5′-UTR, named PEG10-RF1a^His^ (RF1a^His^), PEG10-RF1b^His^ (RF1b^His^), PEG10-RF1a/2^His^ (RF1a/2^His^) and PEG10-RF1b/2^His^ (RF1b/2^His^). Figure 8A shows a schematic view of the different PEG10 constructs. These constructs and the previously described ^His^PEG10-RF1 (^His^RF1) and ^His^PEG10-RF1/2 (^His^RF1/2) constructs [2] without the 5′-UTR sequence were transfected into HEK293 and COS-1 cells. The expressed proteins were detected by Western blot analysis using an anti-His antibody and PEG10-RF1 specific antibodies [2]. For Western blot data interpretation it should be kept in mind that His-epitope tagging increased the molecular weight for all N-terminal His-tagged PEG10 proteins by about 4 kDa and for all C-terminal His-tagged PEG10 proteins by about 5 kDa in comparison to the corresponding endogenous PEG10 proteins.

Transfection of ^His^RF1 into HEK293 cells led to the expression of a PEG10-RF1 specific protein with an expected size of approximately 50 kDa (Figure 8B). Referred to as the ^His^RF1-specific 50 kDa protein. Surprisingly, in cells expressing RF1a^His^, a prominent PEG10-specific approximately 55 kDa protein, and an approximately 50 kDa protein, matching the size of the ^His^RF1 protein, were detected. The 55 kDa protein is referred to as to be the RF1a-specific protein. Furthermore, cells with the RF1b^His^ construct expressed an approximately 60 kDa protein and in addition, but less, a protein of about 50 kDa. The relative amounts of these proteins according to Western blots were lower than those expressed by ^His^RF1 and RF1a^His^. All proteins were detected by an anti-His antibody as well as PEG10-RF1-specific antibodies, confirming that these proteins are PEG10 specific. In the RF1a^His^ and RF1b^His^ lanes proteins smaller than the 50 kDa proteins were detected by the anti-His antibody. These proteins might represent further processed RF1a and RF1b proteins as known for retroviral *gag* proteins. The 60 kDa protein for RF1b^His^ would match the size of a protein using the new exon 1b AUG start codon for translation. Translation efficiency however, appears to be less compared to the other two constructs. The here for the PEG10 proteins determined molecular weights are only rough estimations and do vary depending on what molecular weight standards were used (see M&M).

Next, we tested the protein expression for the three different RF1/2 constructs (with and without the 5′-UTR sequence). The ^His^RF1/2 construct gave rise to the described RF1 protein product of 50 kDa, an approximately 105–110 kDa RF1/2 −1 frameshift translation product and a 40 kDa product (PEG10-CNF) as previously described [2], [7]. All three proteins were detected with anti-His and PEG10-RF1-specific antibodies (Figure 8B). As shown for RF1a^His^ and RF1b^His^, the presence of the 5′-UTR sequence again changed the translation initiation site (TIS) leading to higher molecular weight proteins than for the ^His^RF1/2 construct. For the RF1a/2^His^ expression construct only a 55 kDa protein was detected also present for RF1b/2^His^ in addition to a more prominent 60 kDa protein. These proteins were only detected with RF1-specific PEG10 antibodies but not with an anti-His antibody because only the −1 frameshift products contain the His-tag at their C-terminus. RF1 proteins or RF1/2 proteolytic cleavage products without the C-terminus lack the His-tag.

Aside from expressing larger RF1 proteins than the ^His^RF1 and ^His^RF1/2 constructs, both constructs, RF1a/2^His^ and RF1b/2^His^, also express larger −1 frameshift proteins of approximately 115 kDa. The RF1a/2 and RF1b/2 proteins were detected with the anti-His antibody whereas the reactivity with the PEG10-RF1-specific antibodies varied and appeared to be much weaker. Additional proteins were detected on anti-His Western blots with sizes ranging between those of RF1 and RF1/2 proteins. These proteins were not seen with the PEG10-RF1-specific antibodies suggesting that these proteins are proteolytic C-terminal cleavage fragments of RF1a/2^His^ and RF1b/2^His^ translation products with intact His-tags. Western blots with lysates of COS-1 cells transfected with the different PEG10 expression constructs showed a similar detection pattern as seen for HEK293 cells (data not shown).

The Western blot data for the 5′-UTR expression constructs demonstrated that its presence changes translation initiation suggesting that a TIS exists upstream of the previously assumed ATG start codon. It appeared as if the 55 kDa protein is the predominant RF1 translation product for the 5′-UTR-RF1a/2 mRNA. We noted that this 55 kDa protein corresponds approximately in size to a protein detected with the PEG10-specific antibodies for mock transfected HEK293 cells, which was not seen for mock transfected COS-1 cells. Thus, we were wondering if this protein corresponds to the endogenous PEG10-RF1 translation product since HEK293 cells express *PEG10* (see Figure 2A and 7C) as do so HepG2 cells. Western blot analyses with the PEG10-RF1-specific SAON1 and SAOP2 antibodies demonstrated the presence of a protein in RF1a/2^His^ transfected HEK293 and COS-1 cells (Figure 8C) that matches the size of the endogenous PEG10-RF1 protein of non-transfected HepG2 and HEK293 cells, detected with the PEG10-RF1-specific antibodies (Figure 8C). The HepG2 and HEK293 endogenous PEG10-RF1 protein is clearly of higher molecular weight than the one expressed from the ^His^RF1/2 construct. However, this ^His^RF1 protein corresponds to another protein of approximately 50 kDa in HepG2 and HEK293 cells, which also reacted specifically with the PEG10 antibodies. These results strongly suggest the existence of a preferentially used start codon upstream of the previously predicted AUG but that in addition, in HepG2 and HEK293 cells the AUG is used for translation initiation too.

### Endogenous PEG10 Protein Translation Starts from a CUG Codon

In order to identify the new TIS upstream of the previously one we looked for a new AUG start codon located in frame with the previous one. However, there is no in-frame AUG triplet in the 5′-UTR sequence suggesting that PEG10 might use a non-AUG start codon. Utilization of non-AUG codons in mammalian mRNAs is observed with increasing frequency and the majority of such non-AUG TISs are CUG codons [29], [30], [31]. For PEG10 we identified an in-frame CUG codon 102 nucleotides (equivalent to 34 amino acids) upstream of the AUG codon. Thus, we hypothesized that this CUG is the major start codon for PEG10 translation. In order to proof this hypothesis by experimental data, the CUG, the AUG and both codon sequences together were changed by site directed mutagenesis creating three new expression constructs named PEG10-RF1a/2^mutATG-His^, PEG10-RF1a/2^mutCTG-His^ and PEG10-RF1a/2^mutATG/CTG-His^.

**Figure 9:**
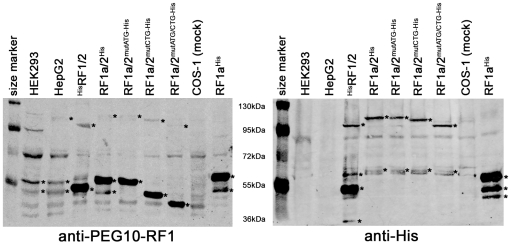
Endogenous PEG10 proteins use non-AUG translation intiation. In order to test whether PEG10 uses a putative non-AUG start codon (CUG) different PEG10 expression constructs (PEG10-RF1a/2^mutATG-His^, PEG10-RF1a/2^mutCTG-His^ and PEG10-RF1a/2^mutATG/CTG-His)^ mutated at the putative translation initiation sites were transfected into COS-1 cells and cell lysates were analysed by SDS-PAGE (8% gel) and Western blot with PEG10-RF1-specific antibodies and an anti-His antibody. Lysates of non-transfected HEK293 and HepG2 cells were analysed in parallel for endogenous PEG10 proteins. Proteins considered to be PEG10-specific are marked by asterisks.

These constructs, as well as the ^His^RF1/2, RF1a/2^His^ and RF1a^His^ constructs, were transfected into COS-1 cells. Cell lysates of transfected cells, mock transfected COS-1 and non-transfected HEK293 and HepG2 cells were analysed by SDS-PAGE and Western blot using the anti-His antibody and PEG10-RF1-specific antibodies. Western blot results are shown in Figure 9 and protein bands considered to be PEG10-specific are marked by an asterisk.

For HEK293 and HepG2 cells several endogenous PEG10 proteins were detected with the PEG10 antibodies. One protein slightly above the 55 kDa reference protein, referred to as the PEG10-specific 55 kDa protein and one below the marker protein, referred to as the PEG10-specific 50 kDa protein (Figure 9, anti-PEG10-RF1 blot). This detection pattern was also observed for RF1a/2^His^ and RF1a^His^. Minor molecular weight differences are due to the His-tag. As expected, only the 50 kDa protein was seen for ^His^RF1/2, which suggests that the 50 kDa protein represents the translation product using the AUG codon. More important for our hypothesis, for cells transfected with RF1a/2^mutATG-His^ only the 55 kDa protein (specific for the CUG start codon) was detected but not the 50 kDa species. Whereas, RF1a/2^mutCTG-His^ transfectants expressed only the 50 kDa protein (specific for the AUG start codon). In accordance with these results, neither the 55 nor the 50 kDa proteins were seen for cells transfected with the double mutant RF1a/2^mutATG/CTG-His^. Surprisingly however, a protein of about 40 kDa reacted specifically with the PEG10 antibodies. Obviously, the cellular translation machinery found a new TIS downstream of the mutated CUG and AUG codons. These data demonstrate that PEG10 indeed uses the CUG as a TIS as well as the AUG leading to PEG10 proteins of different sizes. In addition, -1 frameshift translation appears to be independent of what TIS is selected since all mutant constructs expressed an RF1/2 frameshift product but naturally of different size, which were all detected by the anti-PEG10 antibody and even more specifically by the anti-His antibody (Figure 9). Furthermore, for all RF1/2 constructs (with and without 5′-UTR) proteins of about 60–65 kDa were specifically detected on the anti-His blot most likely representing PEG10 C-terminal (RF1a/2^His^ constructs) or N-terminal (^His^RF1/2 construct) proteolytic cleavage products from the RF1/2 frameshift proteins due to aspartic protease cleavage (see Figure 9 right panel, marked by asterisks). Based on the experience from several Western blots for PEG10 with anti-His and anti-PEG10 antibodies, proteins that were thought to be derived from PEG10 are marked by asterisks in Figure 9.

### *PEG10* and *SGCE* are Co-Expressed in Various Cell Types

**Figure 10:**
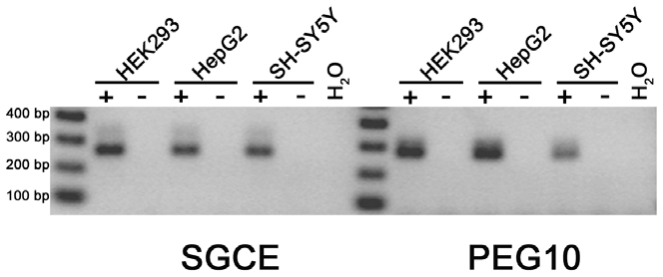
The head-to-head orientated *PEG10* and *SGCE* gene are co-expressed in different cell lines. PCR with the cDNA of HEK293, HepG2 and non-differentiated SH-SY5Y cells and gene-specific primers demonstrates that *PEG10* as well as *SGCE* are co-expressed. +, PCR with cDNA; −, PCR with total RNA.

The *PEG10* and *SGCE* genes are in a head-to-head orientation and separated by less than 200 nucleotides. This region would correspond to the PEG10 −220 promoter region (see Figure 5A). Therefore we were interested to see whether the expression of these two genes is co-regulated. For this purpose we looked for the expression of *PEG10* and *SGCE* in HEK293, HepG2 and SH-SY5Y cells by sqRT-PCR. The results demonstrate that the two genes are co-expressed in all three cell lines. However, it appears that *PEG10* has a higher expression in HEK293 and HepG2 cells but a lower expression in SH-SY5Y cells in comparison with *SGCE* (Figure 10).

## Discussion

Recent reports suggest that PEG10 has important functions in cell proliferation, differentiation, apoptosis and the development of cancer. However, it is not well known how the PEG10 proteins influence these functions and how *PEG10* expression and translation is regulated. In order to understand the molecular mechanism of transcription of a gene, it is essential to identify the corresponding promoter. Determination of the transcription start site (TSS) is the first step in identifying the promoter. Here, we determined what we think is the major TSS (mTSS) and also showed the existence of alternative TSSs (aTSS) further upstream. This is in agreement with studies reporting that many eukaryotic genes do not use a single TSS but that transcription can start from different sites [32]. For example, Suzuki and colleagues analysed 276 human genes and found that the distribution of TSSs is spread over a region of 61.7 bp on average for genes with and without a TATA-box [33]. However, for genes with a TATA-box the TSSs were more tightly clustered. A more recent study performed a genome-wide analysis of mammalian promoters which confirmed the presence of aTSSs for the majority of genes and defined classes of promoters according to the presence and usage of TATA-box, CCAAT-box, GC-box and CpG islands in the context of TSSs [34]. Four categories of promoters and TSSs were classified, (i) a single dominant peak TSS class (SP) with a single dominant TSS, (ii) a general broad TSS distribution (BR), (iii) a broad TSS distribution with a dominant TSS (PB) and (iv) bi- or multimodal TSSs (MU). Based on our data, the PEG10 promoter may belong to the PB class. Two more criteria speak for the here defined PEG10 mTSS. First, it is preceded by a TATA-box (100% consensus sequence) positioned in the ideal distance of 24–30 nucleotides [32]. Second, the sequence around the TSS, G>T(−1)>A(+1)>C>A>C>G, conforms closely to the Py>Py>A(+1)>N>T/A>Py>Py sequence of initiator elements (Inr) [32] in which the initiation site shows the preferred pyrimidine-purine di-nucleotide [35]. The spacing of the TATA-box and Inr would allow these two elements to act synergistically, which is less efficient or neglectable for a distance of more than 30 bp [36]. It is interesting to note, that the sequence of the TATA-box and Inr element and the spacing of the two is highly conserved among various species, i.e. human, mouse, bovine and dog.

We analysed the *PEG10* promoter over a region of 2 kb upstream of the mTSS. Surprisingly, when we sequenced the *PEG10* promoter of 10 unrelated individuals, homozygous and heterozygous deletions of 31 bp (*PEG10* promoter variant 1, PPV1), 37 bp (PPV2) and 71 bp (PPV3) were detected in comparison to the NT_007933|Hs7_8090 reference sequence. These deletions are located 79 bp and 45 bp, respectively, upstream of the mTSS. The deletions represent a low complexity sequence of a CCCCCN repeat. Interestingly, the *Pan troglodytes* and *Macaca mulatta* genomic sequences for this region are 100% identical to the human genome project sequence but a comparison of the CCCCCN repeat region with other mammalian species did not show the repeat. Our data suggest that this part of the human *PEG10* promoter due to its repeat structure represents a region of copy number variation. Unfortunately we were only able to clone and therefore to analyse the PPV1 promoter. Future studies need to investigate the possible effect of copy number variations on *PEG10* promoter activity.

The 1.9 kb PPV1 sequence and shorter versions were cloned into a luciferase reporter and further analysed in HEK293 cells. These cells show endogenous *PEG10* expression and were therefore considered to be well suited for *PEG10* promoter studies. All constructs showed promoter activities and a strong activity was especially seen with the proximal 1 kb promoter region. The lowest promoter activity was seen for the first 844 bp. Higher and approximately equal activities were measured for the proximal 220 and 380 bp of the *PEG10* promoter. The data suggest that positive and negative regulatory regions are spread along the first 1000 bp of the *PEG10* promoter. Negative regulatory sequences may be positioned between nucleotides −380 and −844 and possible positive regulatory motifs are located between nucleotides −844 and −1007 sequences. In addition, further repressor elements might be located between position −1007 and −1910. In conclusion, the *PEG10* −220 proximal PPV1 promoter is sufficient to drive *PEG10* expression and further upstream located in *cis* acting elements modulate *PEG10* expression.

It was recently reported that *PEG10* expression is up regulated by serum deprivation (33). Therefore, we also tested the *PEG10* promoter under low serum concentrations (0.2% FCS). In general, the activity of the promoter constructs were either unchanged or slightly up regulated. In example, serum deprivation led in all assays to a slight but consistently increased PEG10-prom^−1910^ reporter activity (Figure 5B and 5C). Whereas the PEG10-prom^−1007^ reporter showed in the presence of 0.2% FCS either a decreased activity compared to 10% serum conditions (Figure 5B) or a slight increase (Figure 5C) resulting for these experiments in a more or less net null affect. Furthermore, sqRT-PCR analyses for serum deprived HEK293 and HepG2 cells also did not reveal enhanced *PEG10* expression (Figure 6). Thus, our data do not completely agree with the observation that serum deprivation up regulates *PEG10* expression [28]. Nevertheless, in general it can be said that in our assays serum deprivation does not lead to reduced promoter activity. This might be in line with reports that *PEG10* expression increases under cellular stress suggesting that possible stress response elements might be located in the *PEG10* promoter. Such putative stress response elements might be remnants of *PEG10's* lost LTR sequence. LTRs do have promoter functions and are activated upon stress [37]. Increased expression and probably increased amounts of PEG10 proteins might protect the cell from cell death under unfavourable conditions. This would agree with the described anti-apoptotic activity of PEG10-RF1 [16], [20], [21] and that PEG10-RF1 protected L02 hepatocytes from apoptosis after serum deprivation [28].

It was reported that the c-MYC protein up-regulates *PEG10* expression and that there is a positive correlation between increased *c-MYC* and *PEG10* expression in various cancers [19]. Therefore, we tested the possible effect of c-MYC on *PEG10* expression by co-transfecting a *c-MYC* expression construct together with our *PEG10* promoter-reporters into HEK293 cells. Surprisingly, the expression of the c-MYC protein led to an inhibition of all tested promoter constructs independent of the selected serum concentrations. In addition, ectopic c-MYC expression in HEK293 and HepG2 cells did not result increased PEG10 expression. The reason for these different findings is not known but one explanation might be that a c-MYC binding element, a putative E-box sequence located in the *PEG10* intron, was included in the promoter-reporter constructs of this previous study, whereas we analysed only the *PEG10* promoter activity upstream of the mTSS.

In order to identify putative *PEG10*-specific transcription factors and their binding sites in the *PEG10* promoter, the PPV1 sequence was analysed with the Genomatix MatInspector programme. As expected, the TATA-box was identified as a binding site for the TATA-binding protein (TBP). Nucleotide positions −61 to −45 were predicted to be a binding site for E2F members, which was recently experimentally proven [28]. Four more E2F binding sites were predicted in further upstream regions. An SP1 binding site was found at postion −188 to −203. On the (+) as well as on the (−) strand, several sequences were identified related to retroviral regulatory elements like retroviral polyA signals or a lentiviral TATA upstream element. These might be relicts of *PEG10*'s previous retrotransposon nature. In summary, bioinformatic analysis identified a limitied number of putative transcription factors (i.e. TBP, E2F, SP1, AP2) with multiple binding sites for the 1.9 kb *PEG10* PPV1 promoter.

In regard of the −220 proximal *PEG10* promoter, it would be interesting to evaluate if this sequence in a reversed orientation serves as a promoter for the *SGCE* gene. The TBP, E2F and SP1 predicted binding sites for the *PEG10* proximal promoter were also predicted for the opposite strand. The *PEG10* −220 proximal promoter represents the region between the head-to-head orientated *PEG10* and *SGCE* genes. This poses the question whether *PEG10* and *SGCE* are co-regulated due to their shared promoter region. We addressed this question briefly by showing that *PEG10* and *SGCE* are co-expressed in three different cell lines. The results show that transcription of the two genes at the same time is not obscured despite the head-to-head orientation and their overlapping promoter regions. Nevertheless, it appears that among the three tested cell lines the expression level between the two genes varies, suggesting that the overlapping *PEG10* and *SGCE* promoters may be subjected to differential regulation. This is further substantiated by a previous report showing that *PEG10* and *SGCE* are differentially regulated at early pregnancy during the hypoxic phase [14]. In addition, it also might be possible that antisense transcription for *SGCE* or *PEG10*, respectively, could influence the relative levels of the different sense transcripts in certain cells or conditions.

**Figure 11:**
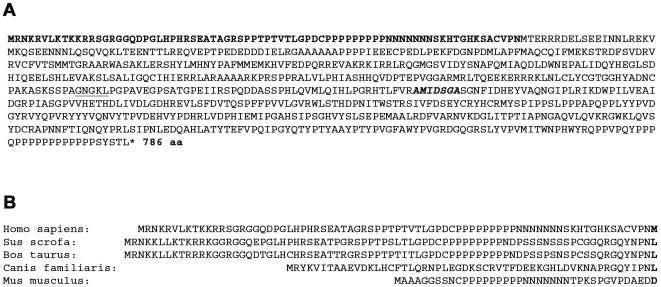
Putative amino acid sequence of the PEG10-RF1b/2 protein and comparison of the N-terminal region with orthologous proteins. A. Shown is the amino acid sequence for the translated PEG10-RF1b/2 protein starting from the putative ATG start codon in exon 1b. The N-terminal part of the human PEG10-RF1b/2 up to the methionine coded by the previously predicted ATG start codon in exon 2 is in bold. The amino acid sequence around the −1 frameshift site is underlined. The aspartate protease motif AMIDSGA is in italic and bold. B. A comparison of the N-terminal part of PEG10 orthologous proteins up to the position corresponding to the human methionine is depicted.

So far, all studies regarding the function of the PEG10 proteins used expression constructs with a coding sequence starting with a predicted AUG start codon in exon 2. This AUG with its surrounding sequence CCCAACAUGA adheres closely to the Kozak sequence, GCCACCAUGG, considered to be optimal as a translation initiation site (TIS) [38]. Our data suggest that due to the presence of the *PEG10* 5′-UTR this AUG codon is not preferentially used as the TIS but that an alternative TIS further upstream exists. This new TIS must be in-frame with the previously assumed AUG start codon and therefore with the C-terminal His-tag, since all products of the C-terminal His-tagged expression constructs were detected with anti-His or anti-PEG10 antibodies. However, this new TIS must represent a non-AUG start codon because there is no in-frame AUG triplet upstream of the previously assumed AUG start codon. Utilization of non-AUG codons in mammalian mRNAs is observed with increasing frequency [29], [30], [31]. The majority of such non-AUG TISs are CUG codons. The VEGF-A gene might serve as one example where protein isoforms exist due to translation initiation at an AUG initiation site but also at an upstream located CUG [30]. Interestingly for PEG10, there is an in-frame CUG codon 102 nucleotides (equivalent to 34 amino acids) upstream of the predicted AUG start codon. Our experimental data unambigiously demonstrate that the defined CUG triplet is indeed used as a translation initiation site. Western blot analysis for endogenous PEG10 proteins in HEK293 and HepG2 cells revealed the existence of several PEG10-specific proteins of different size like i.e. 115 kDa representing the RF1a/2 −1 frameshift product, a 55 kDa protein representing the CUG start site translation product and a 50 kDa protein representing the AUG translation start site product. Interestingly, when we tested an expression construct in which both start sites were mutated we detected a PEG10-specific protein of about 40 kDa (Figure 9). This strongly suggests that there is at least one additional site downstream of the AUG that can be used as a TIS. We do not know whether this additional TIS is also used under normal cell physiological conditions. However, on immunoblots with HepG2 and HEK293 cell lysates a protein of about 40 kDa that reacted with the PEG10 antibodies was occasionally present. Furthermore, we identified a *PEG10* splice variant (PEG10-RF1b/2) that contains a new putative AUG start codon further upstream of the CUG start site and our experimental data (Figure 8B) suggests that this new start site is indeed used by the ribosomal translation complex. Figure 11A shows part of the putative amino acid (aa) sequence for the PEG10-RF1b/2 protein starting from the new in-frame AUG translation codon in exon 1b. This protein is 76 aa longer than the ^His^RF1 protein, which starts with the previously assumed AUG start codon in exon 2.

Translation of pig *PEG10* (DQ779285) starts 237 nucleotides upstream of the previously predicted human AUG and the corresponding 79 amino acids show high similarity (∼80%) to the N-terminal part of human PEG10-RF1b/2. A similar degree of similarity also exists for bovine PEG10 (Figure 11B). Furthermore, comparing human PEG10 with PEG10 from other species, translation of the orthologous proteins starts upstream of the predicted human AUG. For example, the mouse translation initiation site is located 120 nucleotides upstream of the predicted human start codon. In addition, none of the orthologous proteins contain at the human AUG position a methionine (Figure 11B) suggesting that the human CUG TIS is the major translation start site. Thus, depending on what splice variant is expressed and what TIS is selected the translated PEG10 proteins can be about 40 aa or more longer than previously thought. The question now is what function(s) this part of the PEG10 protein might convey?

Further interesting features for *PEG10* were identified. The *PEG10* 5′- and 3′-intron sequence conforms to the canonical consensus splice site sequences GT and AG respectively [39], [40] and the last nucleotide of intron 1 is a consensus G. However, the exon junction sequence is unusual with two As on the exon 2 site instead of the more common ag|GN (N = A, C, G or T) junction motif. mRNA polyadenylation is an essential step for the maturation of almost all eukaryotic mRNAs. Polyadenylation usually occurs at the distal end of the last exon of a gene and the polyadenylation site is preceded by the canonical polyadenylation signal (PAS) A(A/U)UAAA [41] of which two are present in the PEG10 full-length mRNA sequence (see Figure 3). In the literature, it is reported that the canonical PAS was only found for about 75% of analysed transcripts and for about 28% to 49% of genes two or more sites of alternative polyadenylation were identified [42], [43]. Alternative polyadenylation is often observed in a tissue- or time-specific manner [44]. In this study, PEG10 transcripts of different length were detected derived from oligo-dT reverse transcribed RNA. This suggests that alternative polyadenylation might also occur for *PEG10*. Variant polyadenylation uses different PAS motifs instead of the canonical A(A/U)UAAA and several alternative PAS hexamere sequences have been reported [42]. We did not find any of those in the shorter PEG10 transcripts. In case PEG10 transcripts are truly subjected to alternative polyadenylation then future studies have to address the question whether this influences PEG10 expression, mRNA stability, mRNA localisation or translation and if it might be related to disease processes. *PEG10* is most likely derived from a retrotransposon. Therefore, it is interesting to note that non-conserved poly(A) sites are associated with transposable elements to a much greater extent than conserved ones and that the 3′-end of genes can be and were dynamically modified by transposable elements throughout evolution [45].

*PEG10* promotes cell proliferation [15], [16], blocks cell death after serum deprivation [28] and disease related expression of PEG10 inhibits apoptosis [16], [20], [21]. In addition, to its role in cell growth, it was shown that *PEG10* is expressed in stem cells [46], [47] and thus, may be involved in the subsequently occurring differentiation processes. For example, Hishida and colleagues showed that PEG10 promotes the mitotic clonal expansion of adipocytes necessary for adipocyte differentiation and that siRNA mediated PEG10 knock-down blocks the early adipocyte differentiation process [48]. We reported here that the retinoic acid-induced *in vitro* differentiation of the neuroblastoma cell line SH-SY5Y leads to a continuous increase of *PEG10* expression over a period of 14 days. Since *PEG10* is highly expressed in brain PEG10 may also play a role in neuronal cell differentiation.

In this study, we determined the PEG10 major TSS, which allowed us to identify part of the PEG10 promoter and led to the cloning of different promoter-reporter constructs. These constructs can now be used for future studies to investigate how PEG10 expression is regulated. The identification of new PEG10 TISs and that a non-AUG start codon is most likely the major translation initiation site adds new aspects to the already interesting feature of PEG10's –1 frameshift translation mechanism. It is now important to unravel the cellular functions of the PEG10 protein variants and how they are related to normal or pathological conditions. In summary, our study presents new data on the genomic organization as well as expression and translation of *PEG10*, a prerequisite in order to study and understand the role of PEG10 in cancer, embryonic development and normal cell homeostasis.

## Materials and Methods

### Cell Lines and Cell Culture

The following cell lines were used in this study, COS-1, HEK293, HepG2, HL60, HMEC-1, primary HUVECs, K562, SH-SY5Y, SW403, SW948 and T47D. COS-1, HEK293 and SW948 cells were grown in DMEM High Glucose (4,5 g/L) medium with L-Glutamine supplemented with 10% fetal calf serum (FCS) and 100 units Penicillin/Streptomycin (PAA Laboratories). HepG2, HL60, K526, LCLC-103H, SW403 and T47D cells were cultured in RPMI 1640 medium with L-Glutamine supplemented with 10% FCS. SH-SY5Y cells were cultured in Ham's F12/DMEM with L-Glutamine supplemented with 15% FCS. SH-SY5Y cells were differentiated over a period of 14 days with 10 µM *all-trans* retinoic acid. In brief, medium was exchanged 24 hours after seeding for fresh medium containing retinoc acid. This was repeated every 48 hours. HMEC-1 cells were maintained in Endothelial Basal Medium with Endothelial Supplement and 5% FCS. Primary HUVECs were cultured in M199 containing HEPES (16 mM), heparin (6.4 U/ml), gentamicin (80 µg/ml), PD-ECGF (20 µg/ml), 10% foetal calf serum (FCS) and 10% human serum (a kind gift from the Blood Center, Mannheim, Germany) and 20 µg/ml ECGF. All cell lines were cultured at 37°C in a 5% CO_2_ environment. All media and FCS were obtained from PAA Laboratories.

### Sequencing

Sequencing was done on an ABI Prism™ 310 Genetic Analyzer (Applied Biosystems) with gene- or vector-specific primers and the BigDye Terminator v1.1 Cycle Sequencing Mix (Applied Biosystems) according to the manufacturer's instructions.

### 5′-RACE

Total RNA was prepared from the cell lines HepG2 and SH-SY5Y using the *SV Total RNA Isolation System* (Promega) according to the manufacturer's instructions. In order to get the full length 5′-end of the PEG10 transcript the 5′ RACE System for Rapid Amplification of cDNA Ends (Version 2.0, Invitrogen) was used according to the manufacturer's instructions. In brief, first strand cDNA was synthesized by the reverse transcriptase SuperScript™ II from ∼600 ng of total RNA using 2.5 pmol of the *PEG10* gene-specific primer KIAA-3′OUTER. After first strand cDNA synthesis, the mRNA template was removed by treatment with an RNase mixture of RNase H and RNase T1. Unincorporated dNTPs, primers, and proteins were removed from cDNA using a S.N.A.P. column. A homopolymeric tail was then added to the 3′-end of the cDNA using TdT and dCTP. Since the tailing reaction was performed in a PCR-compatible buffer, the entire contents of the reaction could be directly used for a subsequent PCR. PCR amplification was accomplished using Platinum *Taq* DNA polymerase (Invitrogen), a nested, *PEG10*-specific primer, KIAA-3′INNER, and a deoxyinosine-containing anchor primer (AAP) provided with the system. A dilution of the original PCR was re-amplified using the AUAP primer (provided with the system) and *PEG10*-specific primers KIAA-3′INNER or ABP-R2, respectively. 5′ RACE products were cloned into the pCR2.1 TA vector (Invitrogen) for subsequent characterization by restriction digest and sequencing. Inserts were sequenced with vector-specific T7 primer.

### RNA Isolation, Reverse Transcription and RT-PCR

Total RNA of the different cell lines was isolated with QIAshredder and the RNeasy Kit (Qiagen). For reverse transcription (RT) of RNA into cDNA, 1 µg of total RNA was mixed with 2 µl of 10x RT Buffer, 2 µl of dNTP mix (5 mM, each), 2 µl of oligo-dT primer/random hexamers (50 pmol, each), 2 µl of 5 mM DTT, 1 µl of M-MuLV Reverse Transcriptase (200 units/µl, New England Biolabs) and added up to a total volume of 20 µl with RNase-free H_2_O. For the RT minus control no M-MuLV Reverse Transcriptase was added. The mix was inbubated at 37°C for 1 h and enzyme was heat-inactivated at 65°C for 10 min. Afterwards, the volume was filled-up to 50 µl with RNase-free H_2_O. Products were stored at −20°C until use.

For semi-quantative RT-PCR 1 µl of cDNA was used with the indicated *PEG10-* and *GAPDH*-specific primers for gene expression analyses. *GAPDH* served as the endogenous control gene to allow for normalisation between different RNA samples. RT-PCR experiments with subsequent cloning of the PCR products were carried out in 50 µl volumes using Phusion Polymerase (Finnzymes). All RT-PCRs were done in a Techgene cycler (Techne). Cycle conditions were 45 s at 96°C for initial denaturing followed by 25 or 30 cycles at 96°C for 30 s, 58–72°C (primer specific annealing temperature) for 30 s and 72°C for 1 min and a final extension step at 72°C for 5 min.

### Alternative Polyadenylation

For determination of alternative polyadenylation sites the 3′RACE method was used. In brief, RNA was reverse transcribed by using an oligo-dT-Clamp primer (200 pmol). 1 µl cDNA was used as template in a PCR reaction with PEG10 specific forward primers in combination with the Clamp reverse primer. Otherwise the experiment was carried out as described in the preceeding paragraph. PCR products were subsequently cloned into the pPCR-Script Amp SK(+) plasmid and insert sequences were determined by sequencing.

### PEG10 Promoter Cloning

According to the major transcription start site determined in this study, an approximately 2 kb promoter fragment was amplified from genomic DNA isolated from peripheral blood mononuclear cells (PBMNC) with *Pfu* DNA polymerase (Stratagene) and primer pair combination ^1941^PEG10-fwd/^+19^PEG10-rev. The PCR product was cloned into the XhoI/HindIII sites of the multiple cloning site of the luciferase reporter plasmid pGL2-basic (Promega). The promoter sequence was analysed by sequencing. The obtained promoter construct was named PEG10-prom^−1910^. Smaller promoter fragments were generated by PCR using PEG10-prom^−1910^ as template DNA and the ^+19^PEG10-rev primer in combination with different forward primers (^−1666^PEG10-fwd, ^−1555^PEG10-fwd, ^−1007^PEG10-fwd, ^−844^PEG10-fwd, ^−380^PEG10-fwd, ^−220^PEG10-fwd). The underlined nucleotides in the primer sequences (see Table 1) differ from the template sequence and were changed in order to generate an XhoI restriction site. The PCR products were cloned into the XhoI/HindIII sites of the pGL2-basic plasmid resulting in the following PEG10 promoter reporter constructs: PEG10-prom^−1666^, PEG10-prom^−1555^, PEG10-prom^−1007^, PEG10-prom^−844^, PEG10-prom^−380^ and PEG10-prom^−220^.

**Table 1:** List of primers used in this study.

Primer name	Primer sequence
GAPDH-fwd	5′-ACCACAGTCCATGCCATCAC-3′
GAPDH-rev	5′-TCCACCACCCTGTTGCTGTA-3′
G6PD-fwd	5′-GCAAACAGAGTGAGCCCTTC-3′
G6PD-rev	5′-GGGCAAAGAAGTCCTCCAG-3′
Oligo-dT-Clamp	5′-GGCGACGCGTCGACTAGTGCGGCCG-dT_18_N-3′
Clamp reverse	5′- GGCGACGCGTCGACTAGTGCGGCCG-3′
KIAA-3′OUTER,	5′-GAGCTTCTGCACCTGGCTCTG-3′
KIAA-3′INNER,	5′-GTTGTTGATCTCTTCAGAGAGCTC-3′
ABP-F2	5′-CAGAGGAGTCCTCGCGTG-3′
ABP-F2b	5′-CTCGCGTGGTGAGTATGCG-3′
ABP-R2	5′-GGATGGAGGCCTGGATCC-3′
^−1910^PEG10-fwd	5′-ctcgagAATTTGACAGCGGTCACCAG-3′
^−1666^PEG10-fwd	5′-TTGTTTATTCCTCGAGAGGCTCTC-3′
^−1555^PEG10-fwd	5′-TACTAAAATGTGCTCGAGTTTGCTCT-3′
^−1007^PEG10-fwd	5′-TCTGGCCTCGAGCCG-3′
^−844^PEG10-fwd	5′-TTTTGTGTTCTCGAGCACTATCAAG-3′
^−380^PEG10-fwd	5′-GGGACCTCGAGGTCGC-3′
^−220^PEG10-fwd	5′-CCGTCCTCGAGTCTCCC-3′
^−173^PEG10-fwd	5′-CCTCGGTAATCCCGTACTC-3′
^−70^PEG10-fwd	5′-GAGCACGCTGGGATTTGG-3′
^−45^PEG10-fwd	5′-CTCCTCGGTGCAACCTATAT-3′
^+19^PEG10-rev	5′-aagcttCCGAAGTTGAAGCGCGTGT-3′
^+1^PEG10-fwd	5′-ctcgagACACGCGCTTCAACTTCG-3′
PEG10-RF1-rev	5′-aagcttAGCGTAGTGACCTCCTGTTCC-3′
PEG10-RF1/2-rev	5′-aagcttACAGGGTACTGTAAGATGGAGGC-3′
PEG10^mutATG^-fwd	5′-GTGTGTCCCCAATTTGACCGAACGAAG-3′
PEG10^mutATG^-rev	5′-CTTCGTTCGGTCAAATTGGGGACACAC-3′
PEG10^mutCTG^-fwd	5′-CAACCGTCACCTTAGGTCCCGACTG-3′
PEG10^mutCTG^-rev	5′-CAGTCGGGACCTAAGGTGACGGTTG-3′
c-MYC-fwd	5′-tggaattcATGCCCCTCAACGTTAGCTTC-3′
c-MYC-rev	5′-cgctcgagTTACGCACAAGAGTTCCGTAG-3′
c-MYC-fwd2	5′-AGGCTATTCTGCCCATTTGG-3′
c-MYC-rev2	5′-CCACATACAGTCCTGGATGA-3′
SCGE-fwd	5′-ATCACATCGGCCCTAGACAG-3′
SCGE-rev	5′-ACTTCCTGATAGGTGGACAC-3′

### Expression Constructs and Cloning

For cell-type specific expression and Western blot analysis, amino terminal or carboxy terminal His-tagged PEG10 expression constructs and a *c-MYC* expression construct were cloned. ^His^PEG10-RF1 (^His^RF1) and ^His^PEG10-RF1/2 (^His^RF1/2) were previously described [2]. In order to generate PEG10 expression constructs containing the 5′-UTR sequence, the following primer pairs were used ^+1^PEG10-fwd/PEG10-RF1-rev and ^+1^PEG10-fwd/PEG10-RF1/2-rev, respectively (see Table 1), to amplify the RF1 or RF1/2 coding sequence plus the 5′-UTR from HepG2 or SH-SY5Y cDNA. The PCR products were cloned into pcDNA3.1/V5-His-TOPO-TA resulting the following constructs: PEG10-RF1a^His^ (RF1a^His^), PEG10-RF1b^His^ (RF1b^His^), PEG10-RF1a/2^His^ (RF1a/2^His^) and PEG10-RF1b/2^His^ (RF1b/2^His^). RF1b^His^ and RF1b/2^His^ represent splice variants (see Results). *c-MYC* was amplified with the primer pair c-MYC-fwd/c-MYC-rev (see Table 1) from a pool of reversely transcribed total RNA representing different tissues. The *c-MYC* PCR product was cloned into pcDNA4/HisMax-TOPO-TA. All PCRs were done with the Phusion™ High-Fidelity DNA Polymerase (Finnzymes) and all constructs were verified by sequencing.

To analyse the sequence around the splice site in more detail, exon-intron-exon-spanning primers ABP-F2 and ABP-R2 were used to amplify this region from HepG2 and SH-SY5Y cDNA. PCR products were subsequently cloned into pCR2.1 (Invitrogen) by TA-cloning and sequenced. Plasmid DNA from transformed bacteria were also analysed by PCR using splice site specific primer pairs ABP-F2/ABP-R2 (regular splicing) and ABP-F2b/ABP-R2 (alternative splicing).

### Site Directed Mutagenesis

In order to analyse the putative PEG10 translation initiation sites the sequence of the suspected CTG start codon and the previously assumed ATG start site were changed in the PEG10-RF1a/2^His^ expression construct by site directed mutagenesis (Stratagene) according to the manufacturer's instruction. The ATG start site was changed using the PEG10^mutATG^-fwd/PEG10^mutATG^-rev primer pair. The putative CTG start site was changed using the PEG10^mutCTG^-fwd/PEG10^mutCTG^-rev primer pair. The correct sequence change was verified by sequencing.

### Transient Transfections and Western Blots

COS-1 or HEK293 cells were cultured in 6-well plates to 70–80% confluency. For transfections, 4 µl JetPEI (Polyplus Transfections)/2 µg DNA/well were used according to the manufacturer's instructions. Briefly, cells were transfected either with 2 µg empty vector (mock) or with equal molar amounts of different PEG10 expression constructs added up to 2 µg of DNA with empty vector. Two days post transfection, cells were lysed in 400 µl lysis buffer [50 mM Tris-HCl (pH 7.5), 150 mM NaCl, 0.5% NP-40, 50 mM NaF, 1 mM PMSF+Protease Inhibitor Cocktail (Sigma)] at 4°C for 30 min. Approximately 40 µl of total cell lysate were separated by SDS-PAGE and blotted onto nitrocellulose membranes (Machery & Nagel) for immunodetection with the indicated primary antibodies and IRDye® secondary antibodies (Rockland or LI-COR Biosciences) for the Odyssey® Infrared Imaging System (LI-COR Biosciences). As protein size standards the following markers from Fermentas Life Sciences were used, the Prestained Protein Molecular Weight Marker (#SM0441), the PageRuler™ Prestained Protein Ladder (#SM0671) and the PageRuler™ Plus Prestained Protein Ladder (#SM1811). It has to be noted that between these three size markers reference proteins with the same stated molecular weight (i.e. 55 kDa) show a different migration. This makes the determination for the precise molecular weights of the here studied proteins difficult.

### Luciferase Assays

Luciferase-based *PEG10* promoter assays were performed with the Steady-Glo System (Promega). HEK293 or HepG2 cells were seeded into 6-well plates and grown to 80% confluency. Cells were transiently transfected with equal molar amounts (approximately 2 µg) of the different PEG10 promoter constructs. The next day cells of one well were split into 8 wells of a 96-well plate. After complete adherence cells were serum starved for 4 hours. Subsequently, medium was exchanged and cells were incubated for 16 hours with 0.2% FCS or 10% FCS. The pEYFP-N1 vector (Clontech) (100 ng/transfection) was always included to serve as a control for transfection efficiency. Luciferase activity was measured with the Lumi-Imager F1™ (Roche Applied Science). All experiments were performed in triplicate and repeated at least twice.
